# Minimal 2'-O-methyl phosphorothioate linkage modification pattern of synthetic guide RNAs for increased stability and efficient CRISPR-Cas9 gene editing avoiding cellular toxicity

**DOI:** 10.1371/journal.pone.0188593

**Published:** 2017-11-27

**Authors:** Megan Basila, Melissa L. Kelley, Anja van Brabant Smith

**Affiliations:** Dharmacon, a Horizon Discovery Group company, Lafayette, Colorado, United States of America; National Institutes of Health, UNITED STATES

## Abstract

Since its initial application in mammalian cells, CRISPR-Cas9 has rapidly become a preferred method for genome engineering experiments. The Cas9 nuclease is targeted to genomic DNA using guide RNAs (gRNA), either as the native dual RNA system consisting of a DNA-targeting CRISPR RNA (crRNA) and a *trans*-activating crRNA (tracrRNA), or as a chimeric single guide RNA (sgRNA). Entirely DNA-free CRISPR-Cas9 systems using either Cas9 protein or Cas9 mRNA and chemically synthesized gRNAs allow for transient expression of CRISPR-Cas9 components, thereby reducing the potential for off-targeting, which is a significant advantage in therapeutic applications. In addition, the use of synthetic gRNA allows for the incorporation of chemical modifications for enhanced properties including improved stability. Previous studies have demonstrated the utility of chemically modified gRNAs, but have focused on one pattern with multiple modifications in co-electroporation with Cas9 mRNA or multiple modifications and patterns with Cas9 plasmid lipid co-transfections. Here we present gene editing results using a series of chemically modified synthetic sgRNA molecules and chemically modified crRNA:tracrRNA molecules in both electroporation and lipid transfection assessing indel formation and/or phenotypic gene knockout. We show that while modifications are required for co-electroporation with Cas9 mRNA, some modification patterns of the gRNA are toxic to cells compared to the unmodified gRNA and most modification patterns do not significantly improve gene editing efficiency. We also present modification patterns of the gRNA that can modestly improve Cas9 gene editing efficiency when co-transfected with Cas9 mRNA or Cas9 protein (> 1.5-fold difference). These results indicate that for certain applications, including those relevant to primary cells, the incorporation of some, but not all chemical modification patterns on synthetic crRNA:tracrRNA or sgRNA can be beneficial to CRISPR-Cas9 gene editing.

## Introduction

The class II CRISPR-Cas system is a bacterial adaptive defense mechanism that has been applied in mammalian cells for genome engineering [1–4]. Using a small RNA targeting sequence, the *Streptococcus pyogenes* endonuclease Cas9 can bind DNA sequences upstream of an NGG protospacer adjacent motif (PAM) and cause a double-strand break (DSB). When a DSB occurs in mammalian cells, it is repaired by endogenous cellular mechanisms such as non-homologous end joining (NHEJ) or homology-directed repair (HDR). NHEJ is the predominant repair pathway and results in either perfect resolution of the DSB or imperfect repair with either insertions or deletions (indels) of nucleotides at the break site. The result of this imperfect repair can be an alteration of the downstream gene product, potentially causing a functional gene knockout.

Two different guide RNA (gRNA) configurations can be used by the Cas9 nuclease to target DNA. The native bacterial system utilizes a dual RNA-guided system comprised of a CRISPR RNA (crRNA) and a *trans*-activating crRNA (tracrRNA) [1]. The crRNA contains a 20 nucleotide targeting sequence at the 5’ end and a 22 nucleotide repeat region at the 3’ end that is complementary to the tracrRNA. The native tracrRNA is an 89-mer containing the crRNA complementary region at the 5’ end and secondary structure necessary for binding Cas9 at the 3’ end [5]. The second RNA configuration uses a single guide RNA (sgRNA), which is comprised of both crRNA and tracrRNA fused together by a stem loop in the duplex region creating a long ~ 100-mer RNA [1]. This chimeric strategy was employed to simplify the generation of expression plasmids or *in vitro* transcribed sgRNA molecules for editing in mammalian cells [6,7]. Both RNA configurations efficiently guide Cas9 to specific DNA sites in mammalian cells to cause a DSB [8]. Using the dual RNA system allows for rapid synthesis of gene-specific crRNAs that can be used with a universal tracrRNA, and, importantly, permits high-throughput generation of genome-scale libraries for arrayed screening applications [8–12].

Delivery of the gene editing components into mammalian cells can be achieved with several methods, including lipid transfection and electroporation. Lipid transfection delivers nucleic acids or proteins into cells by complexing them with cationic lipids. Molecules enter the cell and are trafficked through the endosomal pathway and eventually released into the cytoplasm. Electroporation is often used for delivery when cells are difficult-to-transfect with lipids, such as primary cells. When cells are electroporated, small membrane pores are created with an electric charge to allow the entry of molecules into the cytoplasm and/or nucleus. Once the electric current is stopped, the cell repairs the pores with these molecules inside the cell. For each delivery method, both nucleic acids and proteins are susceptible to degradation by endo/exonucleases or proteases present in the cell, although the lipid:nucleic acid complex provides a higher level of protection of the delivered cargo compared to electroporation. Specifically, it has been shown that if gRNAs are co-electroporated with Cas9 mRNA or Cas9 plasmid, gene editing efficiency drastically decreases when the gRNAs are unmodified, likely due to degradation of the gRNA [8,13].

Chemical modification of RNAs has been shown to improve serum stability, increase nuclease resistance, increase duplex bond formation, and reduce immune response [14–17]. Hendel *et al*. tested gene editing efficiencies for three chemical modification patterns on synthetic sgRNA molecules: 2’-O-methyl (M), 2’-O-methyl plus phosphorothioates linkages (MS), and 2’-O-methyl plus 3’ thioPACE (MSP) [13]. Both MS- and MSP-modified sgRNAs produced highly efficient gene editing when co-electroporated with Cas9 mRNA in both K-562 cells and primary human T-cells. In other work, 2’-O-methyl, 2’-fluoro and 2’,4’-constrained ethyl-modifications were added along with a fully phosphorothioated backbone to only the crRNA and transfected 24 hours after a plasmid expressing both tracrRNA and Cas9 [18]. These modified crRNAs were compared to expressed sgRNAs in HEK293T cells and were found to improve activity of the native crRNA up to 75% of the expressed sgRNA, depending on the modification pattern [18]. It was not surprising that the modification of crRNA did not improve editing fully to that of the expressed sgRNA, as the expressed sgRNA is constantly present and continually being transcribed, while a synthetic crRNA is only present in the amount delivered. Additionally, gene editing is efficient when unmodified synthetic crRNA:tracrRNA are electroporated into cells following prior electroporation of Cas9 mRNA, but not when electroporated at the same time [8]. However, when synthetic crRNA and tracrRNA containing three MS modifications on three nucleotides and three phosphate bonds at both the 5’ and 3’ ends (3xMS) are co-electroporated with Cas9 mRNA, significant gene editing is detected using a DNA mismatch detection assay, indicating increased persistence of the gRNA [8].

Recent efforts have highlighted the potential for CRISPR-Cas9 gene editing as a therapeutic tool, particularly using a DNA-free approach with Cas9 mRNA and synthetic gRNA for electroporation into clinically relevant cells such as primary T-cells and hematopoietic stem cells (HSCs) [10,19]. With a DNA-free system, the potential for CRISPR-Cas9-mediated off-targeting is minimized due to the transient nature of both the Cas9 and the gRNA [20]. In order to optimize gene editing using DNA-free CRISPR-Cas9 systems and electroporation, we investigated the effect of different 2’-O-methyl and phosphorothioate linkages (PS 2’-OMe or MS) of both synthetic sgRNA and synthetic crRNA:tracrRNA when co-electroporated into cells with Cas9 mRNA or Cas9 protein. We further characterized these modified synthetic gRNAs in lipid-mediated transfection experiments, comparing the modification patterns for gene editing efficiency and functional gene knockout phenotype. We transfected unmodified and modified gRNAs alone into a stably expressing Cas9 cell line, as well as in co-transfection with Cas9 mRNA and Cas9 protein and reported the modification patterns that resulted in significant increases in gene editing efficiency (> 1.5-fold). Importantly, we identified a minimal chemical modification pattern that provided increased stability of the gRNA and avoided cellular toxicity observed with more highly modified gRNA.

## Materials and methods

### Synthetic guide RNA

All sgRNAs, crRNAs and tracrRNA were synthesized with or without the various modification patterns (S1 Table) on solid-phase support using 2′-bis(acetoxyethoxy)-methyl ether (2′-ACE) chemistry at GE Healthcare Dharmacon [21–23]. Synthetic gRNAs were deprotected and desalted. sgRNAs and tracrRNA were further purified by reverse phase HPLC to greater than 85% purity. Lyophilized gRNAs were resuspended in 10 mM Tris-HCl pH 8.0 (Fisher Scientific Cat #BP1758-500). Edit-R™ predesigned crRNA targeting *PSMD7* (GE Healthcare Dharmacon Cat #CR-009621-01-0010) and *PSMD11* (GE Healthcare Dharmacon Cat #CR-011367-04-0010), Edit-R *PPIB* Synthetic crRNA Control Kit (GE Healthcare Dharmacon Cat #UK-007050-20) and Edit-R crRNA Non-targeting Control #1 or #4 (GE Healthcare Dharmacon Cat #U-007501 or U-007504), or the corresponding synthetic sgRNA with the same DNA target site were used in all experiments. The Edit-R tracrRNA (GE Healthcare Dharmacon Cat #U-002000-50) is a 74-mer RNA based on the published *S*. *pyogenes* tracrRNA [1].

### Cell culture

K-562 cells (ATCC CCL-243) were maintained in RPMI 1640 medium (GE Healthcare Hyclone Cat #SH30096.01) and supplemented with 10% fetal bovine serum (FBS; GE Healthcare Hyclone Cat #SH30071.03), 2 mM L-glutamine (GE Healthcare Hyclone SH3003401), 1 mM sodium pyruvate (GE Healthcare Hyclone Cat #SH30239.01), non-essential amino acids (NEAA) (GE Healthcare Hyclone Cat #SH30238.01) and 10 mM HEPES (GE Healthcare Hyclone Cat #SH30237.01). U2OS (ATCC HTB96) cells were cultured in Dulbecco’s Modified Eagle Medium supplemented with 10% FBS, 2 mM L-glutamine; 0.5 mg/mL G418 (GE Healthcare Hyclone Cat #SV30069.01) was included for Ubi-EGFP U2OS cells. HeLa (ATCC CCL-2) cells were cultured in Dulbecco’s Modified Eagle Medium supplemented with 10% FBS, 2 mM L-Glutamine and 1x NEAA.

### Electroporation

At the time of electroporation, 200,000 to 2 million K-562 cells were collected per reaction and centrifuged at 500 × g for 2 minutes. Cell pellets were washed with DPBS (GE Healthcare Hyclone Cat #SH30264.01) for Cas9 mRNA electroporations and re-centrifuged at 500 × g for 2 minutes. Cells were resuspended in appropriate electroporation buffer at 1 million cells/100 μL. Cells were then mixed with Cas9 mRNA (5 μg) and crRNA:tracrRNA or sgRNA (5.36 μM) or pre-complexed Cas9 protein (150 μM) with crRNA:tracrRNA or sgRNA (3 μM). No pre-annealing of crRNA and tracrRNA was done before electroporations. Electroporations were carried out with either the Lonza Nucleofector™ 2b or 96-well Shuttle™ according to the manufacturers protocol. Experiments were performed 2–4 times with gels from a representative experiment in biological duplicates.

### Lipid transfection

For lipid-based delivery of Cas9 mRNA or protein with gRNAs, U2OS or HeLa cells were plated at 10,000 cells/well and Ubi-GFP U2OS cells were plated at 20,000 cells/well in a 96-well format, one day before transfection. At the time of transfections, 200 ng Cas9 mRNA (GE Healthcare Dharmacon Cat #CAS11195) with crRNA:tracrRNA or sgRNA (25 nM) was complexed with DharmaFECT Duo transfection reagent (GE Healthcare Dharmacon Cat #T-2010) in serum-free medium for 20 minutes. No pre-annealing of crRNA and tracrRNA was done before lipid transfections. Following incubation, full serum medium was added to the lipid complex. Medium from cell plates was removed and replaced with the lipid complex. Cells were placed into incubators at 37°C with 5% CO_2_ for 72 hours. Cas9 protein (25 nM; GE Healthcare Dharmacon Cat #CAS11729) with crRNA:tracrRNA or sgRNA (50 nM) was complexed with DharmaFECT 1 transfection reagent (GE Healthcare Dharmacon Cat #T-2001) in serum-free medium for 20 minutes. Following incubation, serum-free medium was added, medium from cell plates was removed and replaced with lipid complex. After 17 hours, lipid complex was removed from cells and replaced with full-serum medium. Cells were incubated at 37°C with 5% CO_2_ for an additional 55 hours. Lipid transfections were performed 3–4 times with representative images from an average of 3 replicate wells within one experiment.

### Cell viability

Before harvesting transfected cells, cell viability was assessed using a resazurin metabolic assay (Fisher Scientific Cat #S25782). Prewarmed resazurin (25 μL) was added to cells and incubated for 1–1.5 hours. Colorimetric measurement was performed with the Wallac 1420 Victor2 Microplate Reader (Perkin Elmer). Transfected samples were normalized to non-targeting control transfected wells.

### Proteasome assay

Transfections were performed as described above, but in black colored 96-well plates. Seventy-two hours post-transfection, medium was replaced with DPBS and plates were scanned using the EnVision Plate Reader (Perkin Elmer). EGFP was scanned from the bottom of the plate and intensity was normalized to the unmodified gRNA transfections. Medium was then replaced and cells were assessed for viability as described above.

### DNA mismatch detection assay

Seventy-two hours post-electroporation/transfection, cells were analyzed for indel formation. Genomic DNA was collected by direct cell lysis in Phusion HF buffer (Thermo Scientific Cat #F-518L), Proteinase K (Thermo Scientific Cat #EO0491), and RNase A (Thermo Scientific Cat #EN0531) for 1 hour at 56°C and then heat inactivated at 98°C for 5–10 minutes. Genomic DNA surrounding the cleavage site was amplified with primers using Touchdown PCR conditions (S2 Table). Following PCR amplification, PCR amplicons were denatured and slowly re-annealed. For the DNA mismatch detection assay, 10 μL of the PCR amplicon was treated with T7 endonuclease I (T7EI; NEB Cat #M0302L) in 1x NEB Buffer 2 for 25 minutes at 37°C. Reactions were stopped with the addition of 6x DNA loading buffer (60% glycerol, 10 mM Tris-HCl pH 8.0, 120 mM EDTA, 0.15% orange G). Cleavage products were resolved on 2% agarose gels by separation at 80 V for 1 hour 30 minutes. Percent gene editing was calculated by measuring band intensities with ImageJ and using the equation: 1 - √(1 –(b+c)/(a+b+c)) × 100 with (b+c) being the area under the curve of the lower cleaved bands and (a+b+c) being the same and also including the area of the uncleaved PCR product (a) [2].

## Results

**Fig 1:**
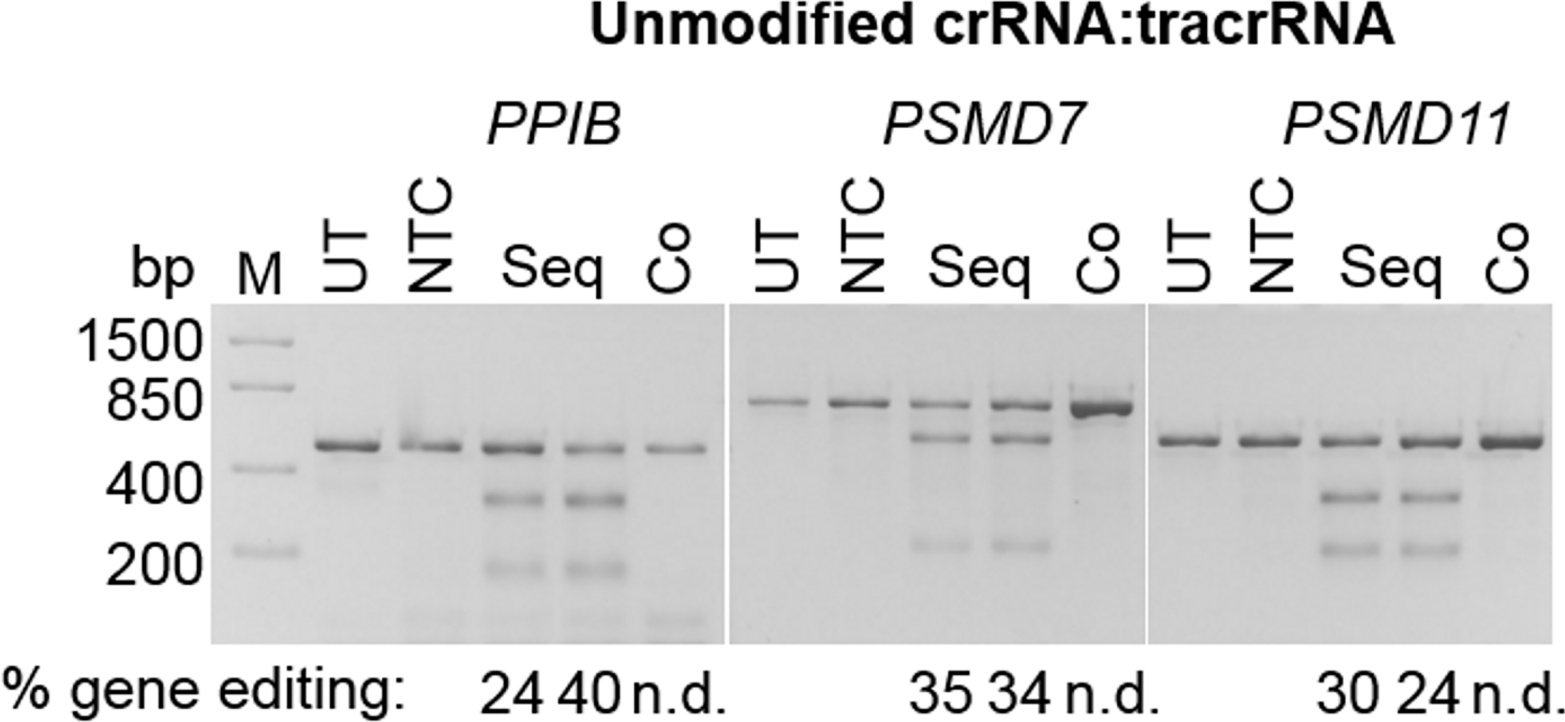
Unmodified crRNA:tracrRNA require a sequential electroporation method with Cas9 mRNA for gene editing. Sequential electroporation involves electroporation of Cas9 mRNA, followed 6 hours later by electroporation of synthetic guide RNA and harvested 2–3 days later for analysis. With a co-electroporation method, both Cas9 mRNA and synthetic guide RNA are delivered into cells at the same time, then harvested 2–3 days later. No detectable (n.d.) gene editing was observed with unmodified synthetic crRNA:tracrRNA in co-electroporation (Co) with Cas9 mRNA into K-562 cells for three gene targets, but resulted in a significant increase when with sequential electroporation (Seq, duplicate samples). UT = Untreated, NTC = Non-targeting control, M = DNA ladder.

In a previous study, we demonstrated that when the unmodified synthetic dual RNA system (crRNA:tracrRNA) targeting *PPIB* was sequentially electroporated following Cas9 mRNA, gene editing was observable in K-562 cells, but in co-electroporation with Cas9 mRNA, gene editing was undetectable [8]. In this study, we further expanded our results with crRNAs targeting genes *PSMD7* and *PSMD11*, as well as including *PPIB*. For sequential electroporations, we first electroporated cells with Cas9 mRNA, then plated these electroporated cells and incubated for 6 hours. Cells were collected and electroporated again with unmodified synthetic crRNA:tracrRNA. Upon sequential delivery of Cas9 mRNA and crRNA:tracrRNA, an average of 32%, 35% and 27% indel formation was estimated by a DNA mismatch detection assay for the gene targets *PPIB*, *PSMD7* and *PSMD11*, respectively (Fig 1). Again, when unmodified crRNA:tracrRNA were co-electroporated with Cas9 mRNA, gene editing was undetectable for all of the gene targets tested (Fig 1). Variability was observed in some replicate sequential electroporation samples, likely due to higher cell death from multiple electroporations within the same day. At the same time, we validated that sequential electroporation was necessary to observe efficient gene editing with Cas9 mRNA and unmodified synthetic sgRNA as previously reported [13]. Upon sequential delivery of Cas9 mRNA and sgRNA, an average of 51%, 28% and 25% indel formation was estimated for the same gene targets *PPIB*, *PSMD7* and *PSMD11*, respectively, while co-electroporation resulted in a drastic reduction in indel formation to ~ 6%, 4% and 16%, respectively (S1 Fig). Overall these data indicate that the shorter crRNAs and tracrRNA are also degraded, potentially at a faster rate than the synthetic sgRNA, since no gene editing was observed for any gene target with co-electroporation. When examining Cas9 protein levels over time after mRNA electroporation, Cas9 protein is detectable by western blot between 4 and 24 hours and is undetectable after 48 hours (panel A in S2 Fig). Additionally, we did not observe a significant difference in Cas9 protein levels between sequentially electroporated versus co-electroporated Cas9 mRNA and gRNAs when examined 24 hours after Cas9 mRNA electroporation (panel B in S2 Fig). This further suggests that the diminished gene editing observed with co-electroporation of Cas9 mRNA and unmodified gRNAs is due degradation of the gRNA and not limited by Cas9 mRNA delivery. Therefore, we tested various chemical modifications to increase the stability of both synthetic gRNA configurations to allow co-electroporation with Cas9 mRNA.

### Minimal chemical modification of gRNAs enable co-electroporation with Cas9 mRNA

**Fig 2:**
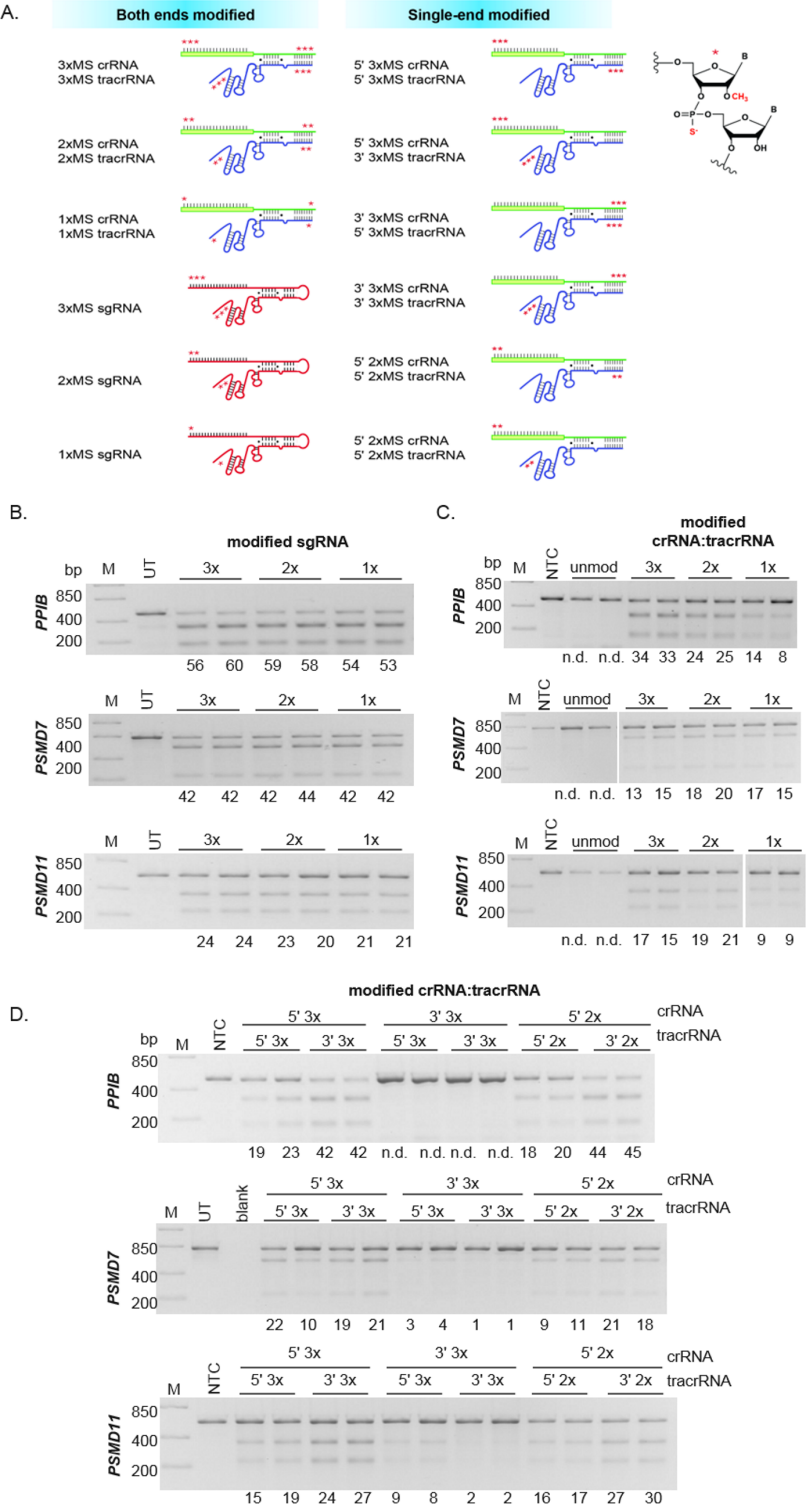
Minimal modification of guide RNA improves stability to increase gene editing efficiency with Cas9 mRNA in co-electroporation. **A.** Modification patterns of synthetic sgRNA (100-mer), crRNA (42-mer) and tracrRNA (74-mer) with one to three 2’-O-methyl modifications with 3’ phosphorothioate linkages (MS, denoted with red *). 1x-3xMS modifications were added to both ends of crRNA, tracrRNA and sgRNA (left column) or the 5’ or 3’ end of crRNA and tracrRNA (right column) and tested in the different combinations shown. Unmodified and modified synthetic guide RNAs targeting genes *PPIB*, *PSMD7* and *PSMD11* were co-electroporated with Cas9 mRNA into K-562 cells (duplicate samples shown; **B-D**). **B.** Co-electroporation of Cas9 mRNA and 1x-3xMS-modified synthetic sgRNA all showed similar levels of gene editing as estimated from a DNA mismatch detection assay. **C.** Unmodified (unmod) crRNA:tracrRNA produced no detectable (n.d.) editing for any of the gene targets, while 1x-3xMS-modified crRNA and tracrRNA had detectable, but varying, levels of gene editing efficiencies. **D.** Single-end modifications on crRNA and tracrRNA indicated that modification of the 5’ end of crRNA is important for stability for efficient gene editing. The numbers under each gel image are percentage of gene editing. UT = Untreated; NTC = Non-targeting control, M = DNA ladder.

To identify the minimal number of chemical modifications required for stability of the synthetic gRNAs, one (1xMS), two (2xMS) or three (3xMS) modifications were applied to both ends of the sgRNA or both ends of synthetic crRNA and tracrRNA and co-electroporated with Cas9 mRNA (Fig 2A). For the sgRNA, when co-electroporated with Cas9 mRNA, all modification patterns resulted in similar levels of gene editing for three gene targets, suggesting that 1xMS on both ends was sufficient to stabilize the long sgRNA (Fig 2B). The addition of any number of modifications produced gene editing levels equivalent to when unmodified sgRNA was sequentially electroporated after Cas9 mRNA (S1 Fig).

With the dual crRNA:tracrRNA, the number of MS modifications on both 5’ and 3’ ends of both molecules did not alter the gene editing and resulted in similar levels of indels for *PSMD7* (Fig 2C). However, for *PPIB* and *PSMD11* targeting, 2x-3xMS modifications were necessary to better stabilize the crRNA and tracrRNA compared to the 1xMS modification as reduced editing (1.8- to 3-fold lower gene editing, respectively) was observed (Fig 2C). This is unlike the 1xMS modified sgRNA results where 1xMS modification provided stability for all three gene targets; this may be because the sgRNA has fewer ends for exonuclease attack compared to crRNA and tracrRNA that each have two ends. Alternatively, 1xMS is not sufficient to protect the most susceptible end of each molecule. As the 1xMS modification on each end crRNA and tracrRNA did not prevent degradation for two of the three genes, no further testing was performed with the 1xMS modifications.

While 2x or 3xMS modifications of both 5’ and 3’ ends on crRNA and tracrRNA provided stabilization to nuclease degradation, we investigated whether modifications of both ends were required to provide the necessary stability. In evaluating the possibilities of modifying crRNA and tracrRNA, MS modifications of the 3’ end of crRNA prevents nuclease degradation of the 3’ overhang while modifications of the 5’ end of tracrRNA stabilize the double-stranded region of the duplex, enhancing hybridization between the RNAs [24,25]. MS modifications on the 5’ end of crRNA and 3’ end of tracrRNA, on the other hand, provide protection of the single-stranded region, and we hypothesized that these modifications may be sufficient to prevent rapid degradation by exonuclease activity.

To test this, we evaluated the editing efficiency of all combinations of 5’ or 3’ modified crRNA and 5’ or 3’ modified tracrRNA with Cas9 mRNA (Fig 2A). Pairing 5’ 2x or 3xMS modified crRNA with 3’ 2x or 3xMS modified tracrRNA showed the highest level of gene editing on average at ~ 43%, 20% and 27% for *PPIB*, *PSMD7* and *PSMD11*, respectively (Fig 2D). 5’ 2x or 3xMS modified crRNA in combination with 5’ 2x or 3xMS modified tracrRNA were on average less efficient for *PPIB* (20%), *PSMD7* (13%) and *PSMD11* (17%; Fig 2D). Little-to-no gene editing was detected for all gene targets with 3’ 3xMS crRNA and 5’ or 3’ 3xMS tracrRNA (Fig 2D). This is not surprising since the duplex regions are more resistant to exonuclease attack and modifying these ends, while leaving the single-stranded region unmodified, likely results in degradation as observed with the unmodified versions of the crRNA:tracrRNA. Due to this reduction in gene editing with the 3’ 3xMS versions of crRNA, the 3’ 2xMS version of crRNA was not tested. Overall, modification of the 5’ end of crRNA and the 3’ end of tracrRNA were required for stabilization as indel formation significantly decreased or was not detected when only the 3’ end of crRNA was modified. Incorporation of 2xMS at these ends appeared sufficient for the enhanced editing efficiency.

### Co-electroporation with modified gRNA and Cas9 protein may improve gene editing activity

Unlike with Cas9 mRNA, delivery of Cas9:gRNA ribonucleoprotein (RNP) complex using electroporation does not require gRNA modifications to generate indels in gene editing experiments [20,26,27]. To determine if the MS modifications could further increase the editing efficiency of sgRNA and/or crRNA:tracrRNA complexed with Cas9 protein, we evaluated indel formation when gRNAs were modified with 1x-3xMS modifications on both ends or with single-end modifications.

**Fig 3:**
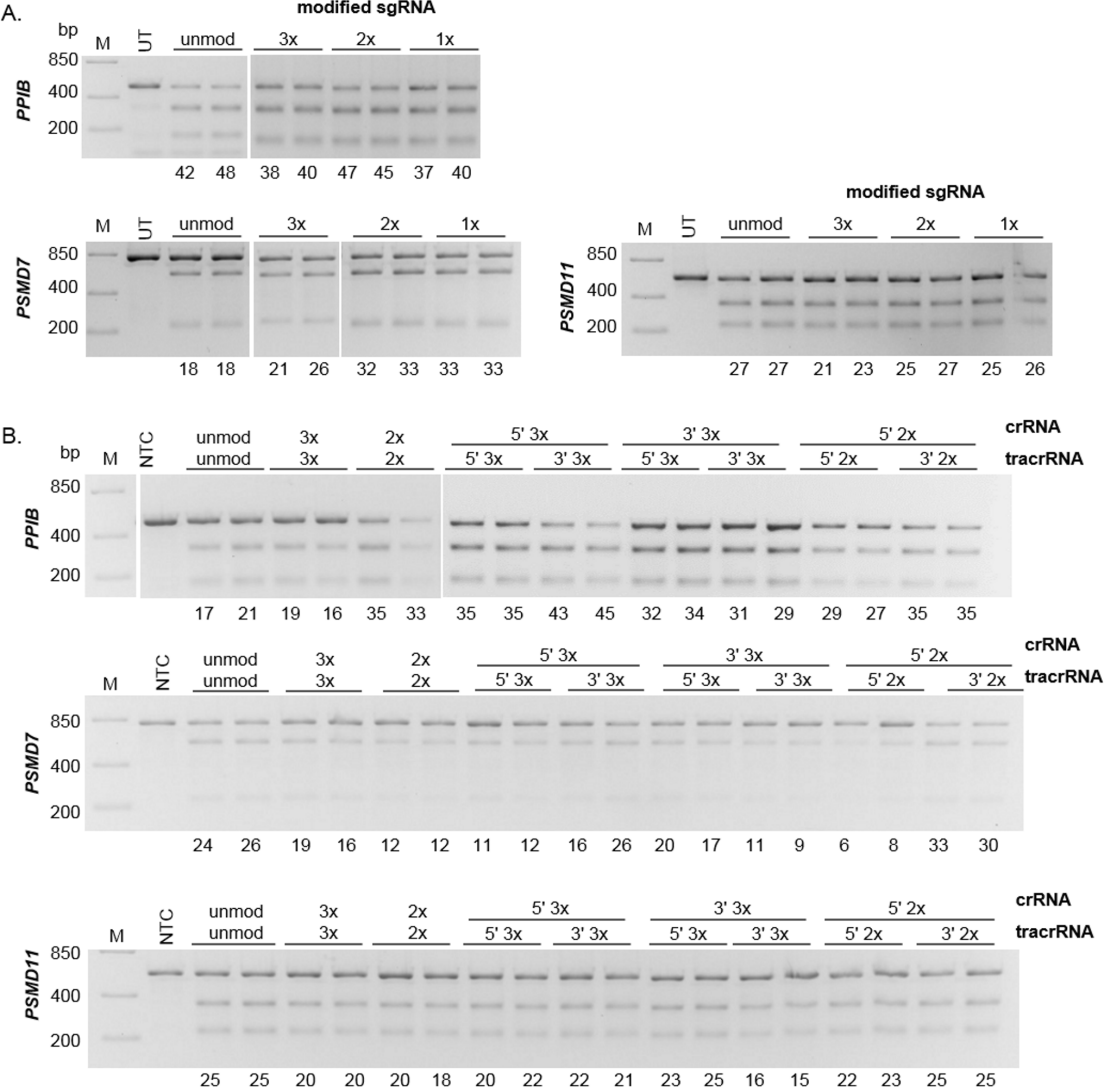
Addition of MS modifications on guide RNAs may improve gene editing efficiency with Cas9 protein in co-electroporation of RNPs. Cas9 protein and unmodified or modified synthetic guide RNAs targeting *PPIB*, *PSMD7* and *PSMD11* were complexed and delivered as RNPs into K-562 cells using co-electroporation (duplicate samples shown). **A.** For most modification patterns, similar gene editing efficiencies were detected with both ends modified sgRNA when compared to unmodified (unmod) sgRNA; only one gene (*PSMD7*) showed increased (1.8-fold) gene editing with 1x-2xMS modifications. **B.** Co-electroporation of both ends and single-end modified crRNA:tracrRNA for genes *PSMD7* and *PSMD11* with Cas9 protein did not result in consistent increase or decrease in gene editing efficiencies compared to unmodified, but increased gene editing with some modification patterns targeting *PPIB*. The numbers under each gel image are percentage of gene editing. UT = Untreated, NTC = Non-targeting control, M = DNA ladder.

For co-electroporations, Cas9 protein was incubated with synthetic gRNAs 10–15 minutes prior to delivery into K-562 cells. With unmodified sgRNAs, ~ 45%, 18% and 27% gene editing was observed for *PPIB*, *PSMD7* and *PSMD11*, respectively (Fig 3A). Similar editing was detected for all other modification patterns except for *PSMD7* where an increase in indel formation was observed with 1x and 2xMS modified sgRNAs, a 1.8-fold increase over unmodified (Fig 3A). This is lower than what was previously reported with 3xMS modified sgRNAs where a 2.3- to 3.8-fold increase in gene editing efficiency for the gene target (*IL2RG*) was observed [13]. The fact that only one out of three gene targets had modestly increased editing efficiency with modified gRNA suggests that increased editing efficiencies with RNP complexes may be target specific.

Because we only saw an increase in gene editing efficiency for one gene target with sgRNA modifications, we predicted modifications of crRNA and tracrRNA would produce similar results with Cas9 protein. Comparing editing efficiencies between unmodified crRNA:tracrRNA and the both ends or single-end modification patterns, no significant improvement (< 1.5-fold increase) in editing efficiencies were observed for *PSMD7* and *PSMD11* gene targets (Fig 3B). Additionally, for *PSMD7*, some of the modification patterns showed lower editing efficiencies when both 5’ ends of crRNA and tracrRNA were modified (Fig 3B). For *PPIB*, there was an modest increase in gene editing (> 1.5-fold) with 5’ 3xMS crRNA:5’ 3xMS tracrRNA, 3’ 3xMS crRNA:5’ 3xMS tracrRNA and 5’ 2xMS crRNA:3’ 2xMS tracrRNA compared to unmodified crRNA:tracrRNA, and a 2.1-fold increase with 5’ 3xMS crRNA:3’ 3xMS tracrRNA (Fig 3B). As with the sgRNA, one of the three genes demonstrated some editing improvement, although with a different gene target, further suggesting that increased editing with RNP complexes is target and/or reagent dependent.

These data also suggest that the Cas9 protein offers protection of the single-strand region of the crRNA (5’ end). This is particularly evident when only the 3’ end of crRNA was modified (protected) and gene editing levels were comparable to 5’ end modified crRNAs. This was contrasting to 3’ modified crRNAs co-delivered with Cas9 mRNA, which had no observable editing (Fig 2D). Overall, the modification patterns tested on the guide RNAs did not result in consistent improvement of gene editing efficiency when co-electroporated with Cas9 protein, but may offer increased efficiency for some gRNA sequences.

### Certain gRNA modification patterns increase cell death with lipid transfection

**Fig 4:**
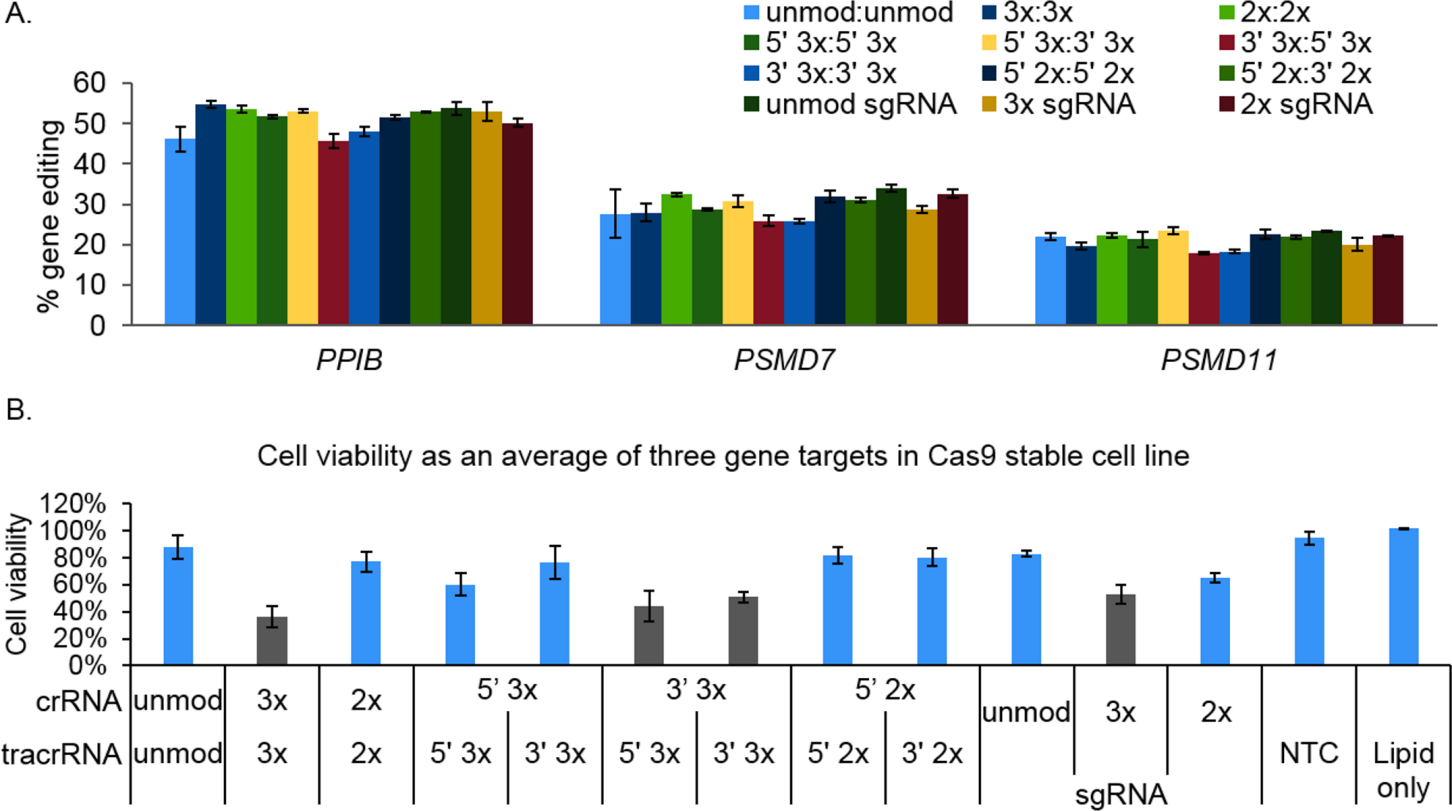
MS modifications of guide RNA produce similar gene editing efficiency to unmodified when lipid transfected into a stably expressing Cas9 cell line and some modification patterns are toxic to cells. **A.** Gene editing efficiency of unmodified (unmod) and modified crRNA:tracrRNA or sgRNA showed similar levels of gene editing efficiencies (< 1.5-fold difference) for each gene when transfected into a stably expressing Cas9 U2OS cell line. Error bars are representative of biological triplicates. **B.** Average cell viability of unmodified or modified guide RNAs for all three genes resulted in a significant decrease in cell viability (< 60%) for some modification patterns (gray bars). NTC = Non-targeting control. Error bars are representative of the average of all three genes with the same modification pattern over two (*PPIB*) or three (*PSMD7* and *PSMD11*) independent experiments.

Previous reports suggest that lipid delivery of nucleic acid differs from electroporation in that it does not require modification of gRNAs for functionality in gene editing experiments [11,28]. In lipid delivery, nucleic acid is proposed to be protected by liposomes when entering the cell and therefore not exposed to RNases present in culture medium or the cytoplasm. However, because of the improvements seen in gene editing with Cas9 mRNA in co-electroporations, we examined if any of these modification patterns could increase the stability of the gRNA, and thus improve Cas9 nuclease activity in lipid transfections. To determine if there was a difference in gene editing efficiencies between unmodified and modified gRNAs, independent of any variability from co-delivery with a Cas9 source, synthetic gRNAs were transfected into a U2OS cell line stably expressing Cas9 under a CAG promoter. We did not observed any of the modification patterns on synthetic crRNA:tracrRNA or sgRNA to significantly improve the gene editing efficiencies compared to the unmodified version for all three gene targets at multiple gRNA concentrations (< 1.5-fold difference in indel formation; Fig 4A and panel A and B in S3 Fig). Additionally, gene editing produced with crRNA:tracrRNA was similar to the gene editing observed with sgRNA for each gene target. This suggested that when Cas9 protein is present within the cell before initiating gene editing, the gRNAs are immediately complexed with Cas9 and protected from degradation. Importantly, when these gRNAs were transfected into the Cas9-stable cell line, some of the modification patterns resulted in cellular toxicity. Cell viability was at an unacceptable level (< 60%) for an average of all three gene targets in duplicate experiments when 3xMS modifications were included on both ends of crRNA, tracrRNA and sgRNA, and also with both combinations of 3’ 3xMS crRNA with 5’ or 3’ 3xMS tracrRNA (grey bars, Fig 4B). These modification patterns remain toxic to cells until a concentration of 6.25 nM is transfected while all other modification patterns remain non-toxic at higher concentrations (50 nM) suggesting that the modification pattern is toxic to cells (panel C in S3 Fig). Additionally, the toxicity observed by some modification patterns is unlikely due to RNA delivery as cell viability was unaffected by the lipid-only control and NTC when normalized to untreated cells. Due to the high cellular toxicity, these modification patterns were not included in the remaining experiments.

### gRNA modifications can modestly improve gene editing and phenotype intensity in lipid co-transfection with Cas9 mRNA or Cas9 protein

**Fig 5:**
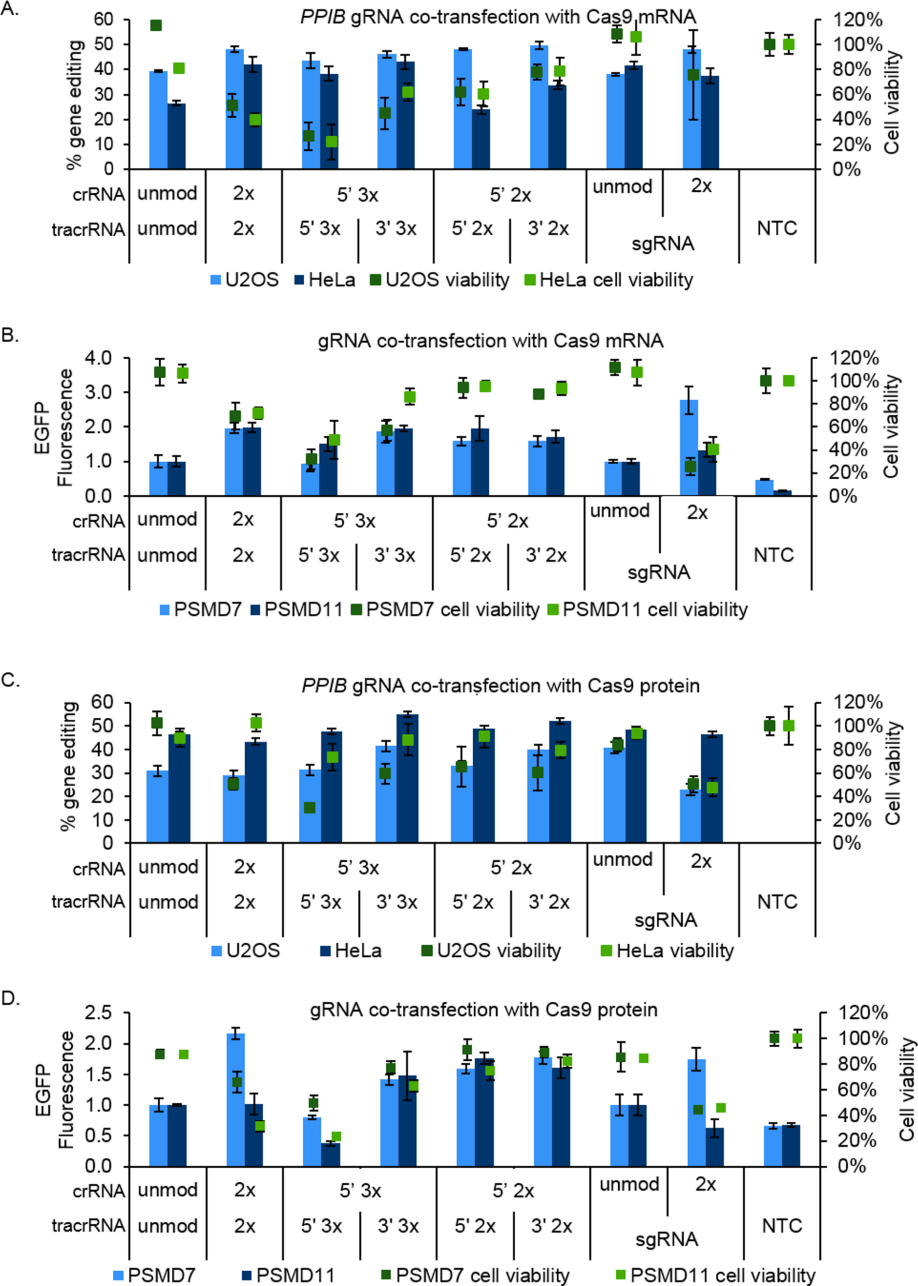
Modest improvement of gene editing efficiency with some MS-modified gRNAs was observed using Cas9 mRNA and Cas9 protein in lipid co-transfections. Unmodified (unmod) and modified guide RNAs were co-transfected with Cas9 mRNA (**A** and **B**) or Cas9 protein (**C** and **D**). Gene editing efficiencies were estimated for *PPIB*-targeting guide RNAs in U2OS cells (light blue bars) and HeLa cells (dark blue bars; **A** and **C**). Functional gene knockout was quantified using a phenotypic analysis with *PSMD7-* (light blue bars) or *PSMD11*- (dark blue bars) targeting guide RNAs in a Ubi-EGFP U2OS cell line (**B** and **D**). Cell viability was assessed for each lipid transfection experiment: U2OS cells (dark green boxes) and HeLa cells (light green boxes; **A** and **C**) and *PSMD7* (dark green boxes) and *PSMD11* (light green boxes; **B** and **D**). NTC = Non-targeting control. Error bars representative of biological triplicates.

While there was no improvement of Cas9 nuclease activity and gene editing efficiency with the modified gRNAs when Cas9 was already present in the cell, we tested whether these modifications could increase indel formation and functional gene knockout when co-transfected with Cas9 mRNA by lipid delivery. We reasoned that for Cas9 mRNA and gRNA in lipid delivery, the RNAs are initially protected by the liposome and stabilization modifications could allow the persistence of the gRNAs once released into the cytoplasm while Cas9 mRNA is translated into protein. Lipid delivery of Cas9 mRNA and gRNAs targeting *PPIB* were tested in two mammalian cell lines (U2OS and HeLa). In U2OS cells, none of the modification patterns increased gene editing efficiency when co-transfected with Cas9 mRNA (< 1.5-fold increase in indel formation; Fig 5A). In HeLa cells, results differed in that only 5’ 2xMS crRNA with 5’ 2xMS or 3’ 2xMS tracrRNA had similar editing as the unmodified crRNA:tracrRNA, while all other modification patterns showed a modest increase in gene editing (> 1.5-fold; Fig 5A). Unmodified sgRNA produced similar levels of indel formation as unmodified crRNA:tracrRNA in U2OS cells, but had higher indels (1.6-fold) in HeLa cells (Fig 5A). Modified sgRNA did not notably increase gene editing (< 1.5-fold) compared to unmodified sgRNA in U2OS cells, and showed no difference in HeLa cells (Fig 5A). In examining the effect of co-transfection of Cas9 mRNA and modified gRNAs, both HeLa and U2OS cell viability was more affected by some modified gRNAs. We observed a significant decrease in cell viability for 2xMS modification on both ends or 5’ 3xMS crRNA with 5’ 3xMS tracrRNA for both cells line using a cutoff of < 60% (Fig 5A). HeLa cells had higher viability (62%) with 5’ 3xMS crRNA and 3’ 3xMS tracrRNA compared to U2OS cells (44%; Fig 5A). Together with the indel formation data, certain modified gRNA patterns can modestly increase the level of indel formation (> 1.5-fold) in some cell lines; however, combining some modified gRNAs with Cas9 mRNA can further decrease cell viability more than with the gRNAs delivered alone.

To observe functional gene knockout, and not just double-strand break and indel repair, a U2OS cell line stably expressing a ubiquitin-conjugated enhanced GFP (Ubi-EGFP) was utilized [29]. In this recombinant cell line, when the proteasome functions normally, ubiquitin-conjugated EGFP is degraded and fluorescence is not observed. By disrupting the function of important proteins in the 26S proteasome through CRISPR-Cas9-targeted gene knockout, EGFP is not degraded and accumulates within the cell to produce a measurable green fluorescent signal [11]. Using this cell line, we tested modified crRNA:tracrRNA and sgRNA targeting *PSMD7* and *PSMD11*, known components of the proteasome, with Cas9 mRNA in lipid co-transfection and compared EGFP fluorescence to the unmodified versions. For each experiment, EGFP fluorescence was normalized to the respective unmodified crRNA:tracrRNA or unmodified sgRNA. Interestingly, all modification patterns modestly increased EGFP fluorescence 1.5- to 2-fold over the intensity measured with unmodified crRNA:tracrRNA for both gene targets, except 5' 3xMS crRNA with 5’ 3xMS tracrRNA for *PSMD7*, which had equivalent levels of gene knockout (Fig 5B). With 5’ 3xMS crRNA and 5’ 3xMS tracrRNA, higher cell toxicity was again observed when targeting *PSMD7* and *PSMD11*. 2xMS modification of synthetic sgRNA increased EGFP fluorescence 2.8-fold over unmodified sgRNA when targeting *PSMD7*, but not significantly for *PSMD11*, and there was an impact on cell viability (Fig 5B). This effect on cell viability could be caused by the disruption of proteasome function as a strong increase in EGFP fluorescence is observed. These results, combined with the indel formation data, suggested that some stabilizing modifications may improve gene knockout efficiency when co-transfected with Cas9 mRNA. However, some patterns may result in nonspecific effects (e.g., increased cell death) that could impact downstream phenotypes of gene editing experiments.

Although we did not observe any consistent increase in gene editing efficiencies when Cas9 protein was co-electroporated with modified synthetic gRNA compared to unmodified, we tested whether modification of the gRNA had any advantages when lipid co-transfected as RNPs. In both U2OS and HeLa cells, all modification patterns had similar cutting efficiencies as unmodified gRNA in co-transfections of the *PPIB*-targeting crRNA:tracrRNAs with Cas9 protein (< 1.5-fold difference; Fig 5C). While unmodified sgRNA and 2xMS sgRNA had similar editing in HeLa cells, the modified sgRNA had lower editing efficiency in U2OS cells (Fig 5C). Cell viability was not compromised in HeLa cells with crRNA:tracrRNA, but some modification patterns (2xMS crRNA:2xMS tracrRNA and 5’ 3xMS crRNA:5’ 3xMS tracrRNA) negatively impacted cell viability in U2OS cells; modified sgRNA in both cell lines showed reduced cell viability (Fig 5C). Overall, co-delivery of Cas9 protein and modified gRNA in lipid transfection did not significantly increase editing efficiencies.

We further tested the modified gRNAs targeting *PSMD7* and *PSMD11* transfected as RNPs for functional gene knockout in the proteasome assay. Single-end modified patterns with 2xMS (5’ 2xMS crRNA with 3’ 2xMS or 5’ 2xMS tracrRNA) showed a modest increase in EGFP fluorescence of 1.6- to 1.8-fold when normalized to unmodified crRNA:tracrRNA without a significant impact on cell viability for both gene targets (Fig 5D). When targeting *PSMD11*, 5’ 3xMS crRNA:3’ 3xMS tracrRNA did increase EGFP fluorescence 1.5-fold, unlike when targeting *PSMD7*, only a 1.4-fold increase was observed (Fig 5D). With 5’ 3xMS crRNA:5’ 3xMS tracrRNA, EGFP fluorescence was similar or decreased to unmodified, but increased cell death was detected (Fig 5D). 2xMS crRNA:2xMS tracrRNA resulted in a ~ 2-fold increase in fluorescence intensity with knockout of *PSMD7*, and EGFP levels similar to unmodified crRNA:tracrRNA were measured with knockout *PSMD11* but with high cellular toxicity (Fig 5D). When unmodified synthetic sgRNA was compared to 2xMS sgRNA, EGFP fluorescence increased 1.8-fold for *PSMD7*; however, a 1.6-fold decrease was observed with *PSMD11* 2xMS sgRNA, and again an effect on cellular toxicity was observed for both gene targets. Overall, 5’ 2xMS crRNA with 5’ or 3’ 2xMS tracrRNA were the only modification patterns that resulted in a modest increase in functional knockout for both gene targets while maintaining cell viability.

## Discussion

In this study, we expanded on previously reported results by systematically evaluating stabilizing MS modifications, which may also improve gene editing efficiency, on chemically synthesized sgRNA, as well as modified synthetic crRNA and tracrRNA. We demonstrated that modifications are essential for the two-part synthetic crRNA:tracrRNA in co-electroporation experiments with Cas9 mRNA (Fig 2C and 2D). When using a Cas9-stable cell line and lipid transfection, modifications were not required for stability and did not enhance gene editing for three gene targets (Fig 4A), suggesting that increased stability is necessary for only some applications. In agreement with a previous report [13], end modifications of the synthetic sgRNA are also required for co-delivery with Cas9 mRNA using electroporation; without modifications, significantly reduced gene editing was observed (S1 Fig), and like crRNA:tracrRNA, modifications were not necessary for increased stability or improvement of indel formation in cells stably expressing Cas9 (Fig 4A).

To determine the minimal number of MS modifications, we varied the number of modifications to achieve adequate stability of the gRNAs for co-electroporation with Cas9 mRNA. Modifying both ends of sgRNA with only 1xMS on each end was sufficient to restore indel formation to similar levels observed when unmodified sgRNAs were sequentially electroporated with Cas9 mRNA (S1 Fig and Fig 2B). However, 1xMS modifications on both ends of crRNA:tracrRNA did not consistently have the highest levels of gene editing efficiency compared to 2x or 3xMS modified crRNA:tracrRNA for two of the three gene targets (Fig 2C). We further showed that modification of the single-stranded region of crRNA and tracrRNA (5’ end of crRNA with 3’ end of tracrRNA) was important for stability and resulted in the overall highest gene editing efficiencies, compared to modifying the duplex region (3’ end of crRNA with 5’ end of tracrRNA; Fig 2D). Based on crystal structures of Cas9 protein complexed with sgRNA, very few protein:RNA interactions occur at the end of the repeat-anti-repeat region near the tetraloop [30]. Since the sgRNA is a truncated version of the native sequence 3’ end of crRNA and 5’ end of tracrRNA connected by the tetraloop, it seems unlikely that modifications at the end of the duplex region of crRNA:tracrRNA are affecting complexing with Cas9 protein. Likely the reason for the significantly lower activity with 3’ modified crRNA is due to the 5’ unmodified, single-stranded targeting sequence being quickly degraded by exonucleases before Cas9 mRNA is translated into protein, thus disrupting DNA targeting by Cas9.

When co-delivering synthetic gRNAs as RNPs, modifications are not required for stabilization as efficient gene editing was detected whether modified or unmodified in electroporation (Fig 3A and 3B). Additionally, we did not observe a consistent increase in gene editing efficiency with modified gRNAs and Cas9 protein (Fig 3A and 3B). A previous study reported that 3xMS modifications on both ends of an *IL2RG-*targeting synthetic sgRNA could improve gene editing efficiency when co-electroporated with Cas9 protein into K-562 cells (2.3- to 3.8-fold increase by TIDE analysis) [13]. They concluded that modification of the sgRNA has an advantage over unmodified with RNP, but they did not test modifications on synthetic crRNA and tracrRNA. In this study, one (*PPIB*) of three gene targets showed a modest increase in indel formation with some of the modification patterns tested for crRNA:tracrRNA and one (*PSMD7*) of three gene targets showed improved gene editing efficiency with 1x or 2xMS modified sgRNA compared to unmodified (1.7-fold and 1.8-fold increase, respectively, using a DNA mismatch detection assay; Fig 3A and 3B). Taken together, modifications can improve gene editing when delivered as RNPs using electroporation, but this may be targeting-sequence specific and not a universal benefit.

In lipid transfections, when Cas9 nuclease is already present within the cell, modifications of the gRNAs did not improve Cas9 editing efficiency, as all modification patterns on crRNA:tracrRNA and sgRNA resulted in similar efficiencies as the unmodified version (Fig 4A), again suggesting that increased stability is not required for all applications. Importantly, the number and placement of modifications can contribute to a significant increase in cytotoxicity. With 3xMS modifications on both ends of synthetic sgRNA and crRNA:tracrRNA, as well as with 3xMS on the 3’ end of crRNA when paired with 5’ or 3’ 3xMS tracrRNA, significant cell death (< 60% cell viability) was observed in Cas9-expressing cells (Fig 4B). One possible cause of this could be due to the higher number of phosphorothioate linkages, which are known to elicit an immune response [31,32]. The modification patterns that were less or nontoxic (> 60% cell viability) typically contained fewer modifications. Additionally, with some of the modified gRNAs, having acceptable cell viability, in lipid co-transfections with Cas9 mRNA or Cas9 protein further exacerbated the negative effect on cell viability (5’ 3xMS crRNA:5’ 3xMS tracrRNA, 2xMS crRNA:2xMS tracrRNA; Fig 5A). However, some modification patterns maintained acceptable cell viability throughout all co-transfection experiments, including 5’ 2xMS crRNA with 5’ or 3’ 2xMS tracrRNA.

In lipid delivery experiments using Cas9 mRNA, for modifications with the lowest cellular toxicity a small increase (1.5–1.6-fold) in indel formation was observed with modified crRNA:tracrRNA targeting *PPIB* in HeLa cell lines (Fig 5A and 5C), suggesting that unmodified gRNAs have sufficient stability to allow time for Cas9 translation. It is important to note that mismatch detection assays measure formation of indels, but underrepresent the actual gene editing in a cell population due to the lack of sensitivity for very small and relatively large indels; these assays also do not predict the functional gene knockout as some indels result in in-frame mutations and an endogenous protein that retains function. Therefore, a phenotypic assay indicates more accurately the functional gene knockout in a population of cells. In this phenotypic proteasome assay, some of the MS modification patterns tested in co-transfections with Cas9 mRNA or Cas9 protein also showed only a modest increase (up to ~ 2-fold) in EGFP intensity, which indicates knockout of the functional protein (Fig 5B and 5D). This further supported that stability modifications on synthetic gRNAs are not necessary for all applications. Although, consistently within the phenotypic assays, a slight increase was achieved with both Cas9 formats and both gene targets utilizing 5’ 2xMS crRNA with 5’ or 3’ 2xMS tracrRNA (1.5- to 2-fold increase, Fig 5B and 5D).

In summary, our systematic evaluation of the placement and number of MS-modified gRNAs demonstrated that for certain CRISPR-Cas9 genome engineering applications, modifications to stabilize the gRNAs are essential, such as for co-electroporation with Cas9 mRNA (Table 1). Other applications do not require modifications and show no improvement in gene editing as we observed when transfecting gRNAs into a stably expressing Cas9 cell line. But in other experiments such as lipid co-transfection with Cas9 mRNA or Cas9 protein, modified gRNAs may result in a modest improvement in gene editing efficiency that is sequence and target specific (1.5- to 2-fold increase; Table 1). Modifications on the single-stranded region of crRNA and tracrRNA, such as with 5’ 2xMS crRNA with 3’ 2xMS tracrRNA, provided sufficient stability for co-electroporation with Cas9 mRNA and resulted in similar or slightly increased gene editing efficiencies. With sgRNA, 1xMS modification on both ends is adequate for stability in co-electroporation with Cas9 mRNA; however, a minimum of 2xMS are required on the crRNA and tracrRNA. This is likely due to crRNA and tracrRNA being two single-strand RNAs that can be degraded from either end, and further investigation would be needed to fully understand this difference. Using a minimal number of modifications is important to consider to avoid cellular toxicity effects, as significant cell death was observed especially with most of the 3xMS modifications in lipid transfection.

**Table 1:** Summary of results from modifying gRNAs in different applications compared to unmodified gRNAs. A comparison of unmodified and modified gRNAs in different applications, from electroporations to lipid co-transfections, based on the results in this paper. N.d. = not determined, * some modification patterns can be toxic to cells.

Application	Editing efficiency	
	Unmodified gRNA	Modified gRNA
Sequential electroporation with Cas9 mRNA	+++	n.d.
Co-electroporation with Cas9 mRNA	-	+++
Co-electroporation with Cas9 protein	++	++/+++
Transfection into Cas9-expressing cells	+++	+++*
Co-transfection with Cas9 mRNA	++	++/+++*
Co-transfection with Cas9 protein	++	++/+++*

As use of synthetic gRNAs continues to gain popularity, there is potential to apply these modification patterns to increase the half-life of synthetic gRNAs for greater efficiencies in other applications and technologies, including CRISPR interference (CRISPRi) and CRISPR activation (CRISPRa), genomic loci labeling, and other CRISPR systems [33–36]. Additionally, a previous report has suggested the use of a highly modified synthetic crRNA with plasmid-expressed Cas9 and tracrRNA could overcome obstacles in therapeutic applications [18]. While the Cas9 and tracrRNA should not be active without the synthetic crRNA, there is still the potential of random integration of plasmid DNA, especially with electroporation [37]. A major benefit to using synthetic gRNA coupled with Cas9 mRNA or Cas9 protein allows for a completely DNA-free CRISPR-Cas9 genome engineering system for many applications. Recent advances have shown the potential of using Cas9 mRNA or Cas9 protein for therapeutic applications, specifically with the use of electroporation or lipids for delivery *ex vivo* or *in vivo* [38,39]. The addition of an optimal chemical modification pattern on the gRNA in these applications will allow for the highest gene editing efficiencies while minimizing cellular toxicity.

## Supporting information

S1 Table Sequences of synthetic sgRNA, crRNA and tracrRNA, including modification locations. All gRNAs used in this study with 2'-O-methyl (m) and Phosphorothioate linkages (*) denoted.
(XLSX)

S2 Table PCR primers for amplification of gRNA target sites from genomic DNA for DNA mismatch detection assay. (TIF)

S1 Fig Unmodified synthetic sgRNA require a sequential electroporation method with Cas9 mRNA for efficient gene editing. Sequential electroporation protocol involved electroporation of Cas9 mRNA, followed 6 hours later by electroporation of synthetic sgRNA and harvested 2–3 days later for analysis. With a co-electroporation method, both Cas9 mRNA and synthetic sgRNA can be delivered into cells at the same time, then harvested 2–3 days later. Lower gene editing was observed with unmodified synthetic sgRNA in co-electroporation (Co) with Cas9 mRNA into K-562 cells compared to sequential electroporation (Seq), which resulted in a significant increase (1.6 to 8.5-fold). UT = Untreated; M = DNA ladder.
(TIF)

S2 Fig Cas9 protein levels are detectable 4 hours after electroporation and are similar regardless of electroporation delivery method. **A.** Cas9 mRNA was co-electroporated with crRNA:tracrRNA in K-562 cells and Cas9 protein levels were examined over time by western blot with *β-Actin* used as a loading control. Within 4 hours after electroporation, Cas9 protein is detectable until 24 hours. At 48 and 72 hours, Cas9 protein is no longer detected. **B.** Cells were electroporated with Cas9 mRNA alone, and then 6 hours later, cells were again electroporated with or without crRNA:tracrRNA (Sequential) and compared to co-electroporations of Cas9 mRNA and crRNA:tracrRNA, with only one electroporation. No difference in Cas9 protein levels were observed by western blot detection 24 hours after Cas9 mRNA electroporation. A stably expressing Cas9 cell line was used as a positive control for Cas9 detection. For all western samples, 500,000 cells were lysed on ice with 50 μL of a RIPA based lysis buffer supplemented with 1x Protease Inhibitor Mix (GE Healthcare, Cat # 80-6501-23). NuPAGE^TM^ 4X LDS sample buffer and NuPAGE^TM^ Sample Reducing Agent (10X) (Life Technologies, Cat #NP0008, # NP0009) were added to samples before gel electrophoresis. Samples were loaded onto a Novex™ 4–20% Tris Glycine Mini Protein Gel (Thermo Fisher Scientific, Cat #EC6025BOX) and ran per the manufacturers protocol. The protein was transferred to a 0.2 μm Amersham Protran nitrocellulose membrane (GE Healthcare, Cat #10600104) using the Invitrogen™ Xcell II Blot Module (Thermo Fisher Scientific, Cat #EI0002). After transfer, the membranes were blocked for 30 minutes in SuperBlock™ (PBS formulation) (Thermo Scientific, Cat #37515). Primary antibody [mouse anti-Cas9 polyclonal 1:500 dilution (Novus Biologicals, Cat #NBP2-36440), or mouse anti-beta-actin 1:2000 dilution (Abcam, Cat #6276)] was diluted in SuperBlock and incubated overnight at 4°C. Membranes were washed and secondary antibody [goat anti-mouse IgG (H+L) Secondary Antibody, HRP conjugate (Thermo Scientific, Cat #32430))] was diluted 1:20,000 in SuperBlock (PBS formulation) with 0.5% Tween20 and incubated with membranes for 2 hours at room temperature. The membranes were washed and then submerged in SuperSignal™ West Dura Substrate (Thermo Scientific, Cat #34016) solution for beta-actin blots and Super West Femto Maximum Sensitivity Substrate (Thermo Scientific, Cat #34095) for Cas9, and exposed to film.
(TIF)

S3 Fig MS modified guide RNAs perform similarly to unmodified in a stably expressing Cas9 cell line with some toxicity observed at higher concentrations. Gene editing efficiency of unmodified (unmod) and modified crRNA:tracrRNA or sgRNA showed similar levels of gene editing efficiencies (< 1.5-fold difference) measured by EGFP fluorescence from knockout of a proteasome component, *PSMD7* (**A.**) or *PSMD11* (**B.**), at multiple concentrations when transfected at 1.5625 nM to 50 nM at 2-fold increments into a stably expressing Cas9 U2OS cell line. Error bars are representative of biological triplicates. **C.** Average cell viability of unmodified or modified guide RNAs for two genes (*PSMD7* and *PSMD11*) resulted in a significant decrease in cell viability (< 60%) for some modification patterns at concentrations higher than 6.25 nM. NTC = Non-targeting control. Error bars are representative of the average of biological triplicates of two genes with the same modification pattern in one experiment.
(TIF)
