# Supplementary figures and images for: Minimal 2'-O-methyl phosphorothioate linkage modification pattern of synthetic guide RNAs for increased stability and efficient CRISPR-Cas9 gene editing avoiding cellular toxicity
Source: PLoS One. 2017 Nov 27;12(11):e0188593. doi: 10.1371/journal.pone.0188593 (PMC5703482; doi:10.1371/journal.pone.0188593)

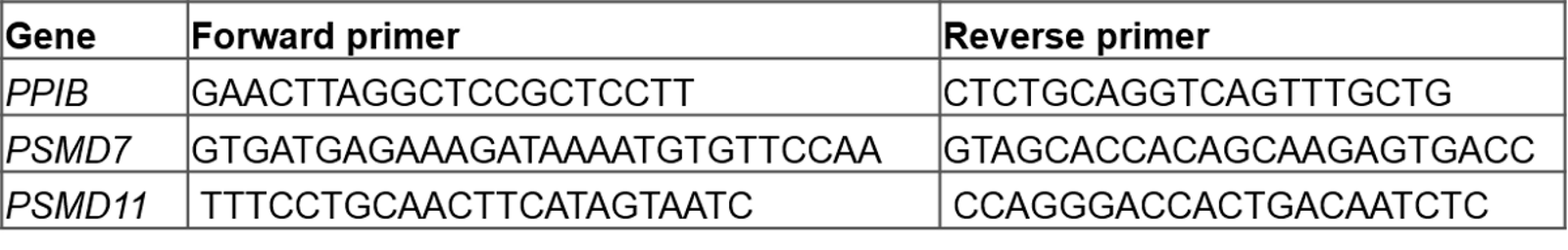

Supplement: S2 Table — (TIF) [file pone.0188593.s002.tif]

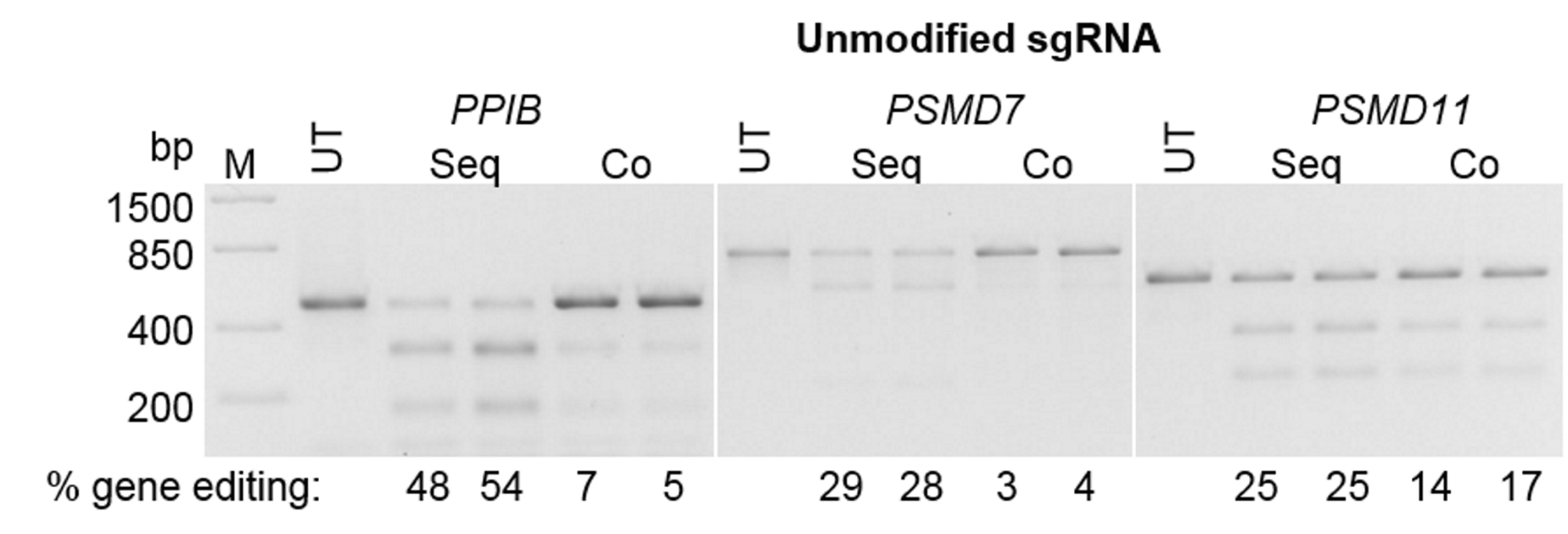

Supplement: S1 Fig — Sequential electroporation protocol involved electroporation of Cas9 mRNA, followed 6 hours later by electroporation of synthetic sgRNA and harvested 2–3 days later for analysis. With a co-electroporation method, both Cas9 mRNA and synthetic sgRNA can be delivered into cells at the same time, then harvested 2–3 days later. Lower gene editing was observed with unmodified synthetic sgRNA in co-electroporation (Co) with Cas9 mRNA into K-562 cells compared to sequential electroporation (Seq), which resulted in a significant increase (1.6 to 8.5-fold). UT = Untreated; M = DNA ladder. (TIF) [file pone.0188593.s003.tif]

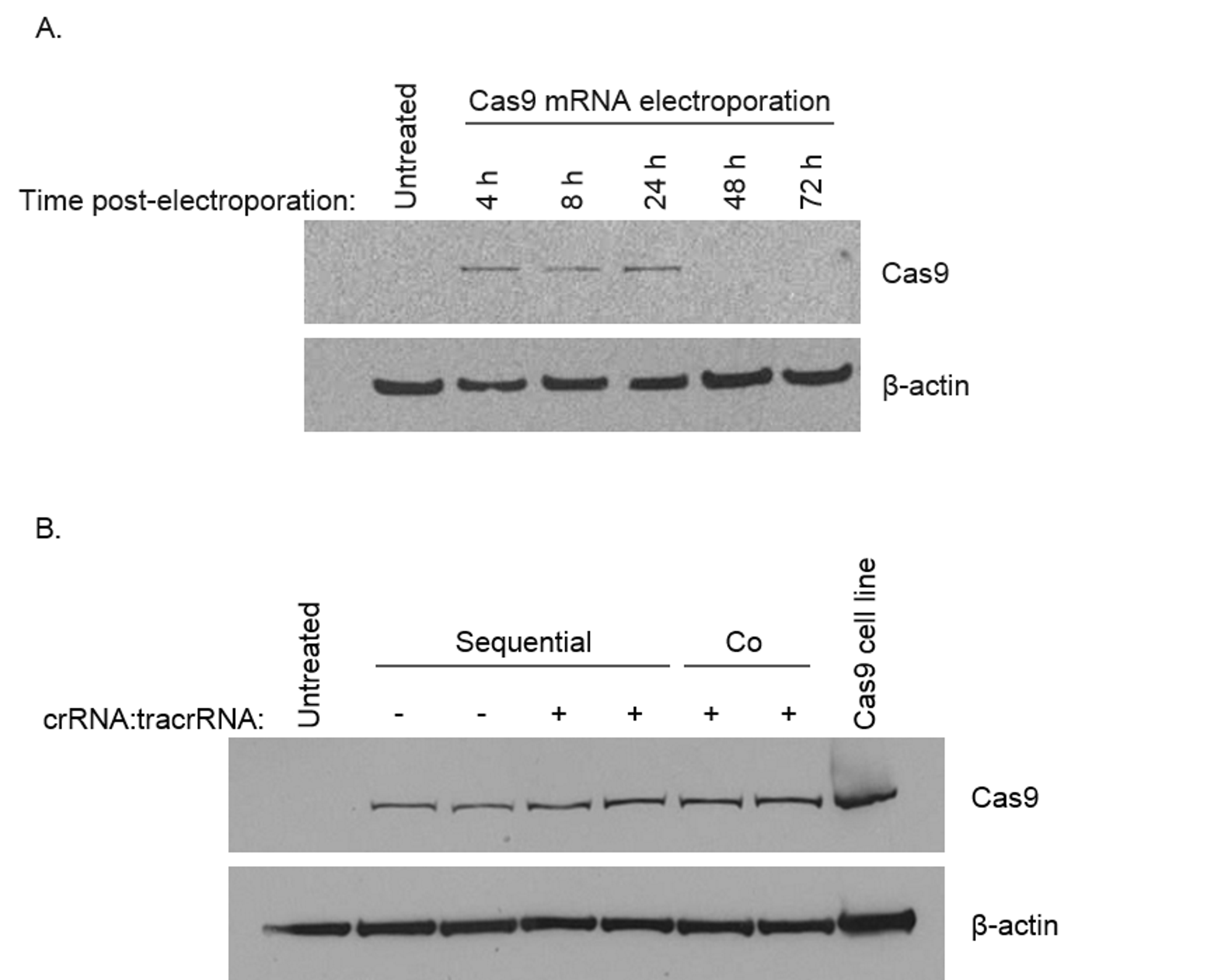

Supplement: S2 Fig — A. Cas9 mRNA was co-electroporated with crRNA:tracrRNA in K-562 cells and Cas9 protein levels were examined over time by western blot with β-Actin used as a loading control. Within 4 hours after electroporation, Cas9 protein is detectable until 24 hours. At 48 and 72 hours, Cas9 protein is no longer detected. B. Cells were electroporated with Cas9 mRNA alone, and then 6 hours later, cells were again electroporated with or without crRNA:tracrRNA (Sequential) and compared to co-electroporations of Cas9 mRNA and crRNA:tracrRNA, with only one electroporation. No difference in Cas9 protein levels were observed by western blot detection 24 hours after Cas9 mRNA electroporation. A stably expressing Cas9 cell line was used as a positive control for Cas9 detection. For all western samples, 500,000 cells were lysed on ice with 50 μL of a RIPA based lysis buffer supplemented with 1x Protease Inhibitor Mix (GE Healthcare, Cat # 80-6501-23). NuPAGETM 4X LDS sample buffer and NuPAGETM Sample Reducing Agent (10X) (Life Technologies, Cat #NP0008, # NP0009) were added to samples before gel electrophoresis. Samples were loaded onto a Novex™ 4–20% Tris Glycine Mini Protein Gel (Thermo Fisher Scientific, Cat #EC6025BOX) and ran per the manufacturers protocol. The protein was transferred to a 0.2 μm Amersham Protran nitrocellulose membrane (GE Healthcare, Cat #10600104) using the Invitrogen™ Xcell II Blot Module (Thermo Fisher Scientific, Cat #EI0002). After transfer, the membranes were blocked for 30 minutes in SuperBlock™ (PBS formulation) (Thermo Scientific, Cat #37515). Primary antibody [mouse anti-Cas9 polyclonal 1:500 dilution (Novus Biologicals, Cat #NBP2-36440), or mouse anti-beta-actin 1:2000 dilution (Abcam, Cat #6276)] was diluted in SuperBlock and incubated overnight at 4°C. Membranes were washed and secondary antibody [goat anti-mouse IgG (H+L) Secondary Antibody, HRP conjugate (Thermo Scientific, Cat #32430))] was diluted 1:20,000 in SuperBlock (PBS formulation) with 0.5% [file pone.0188593.s004.tif]

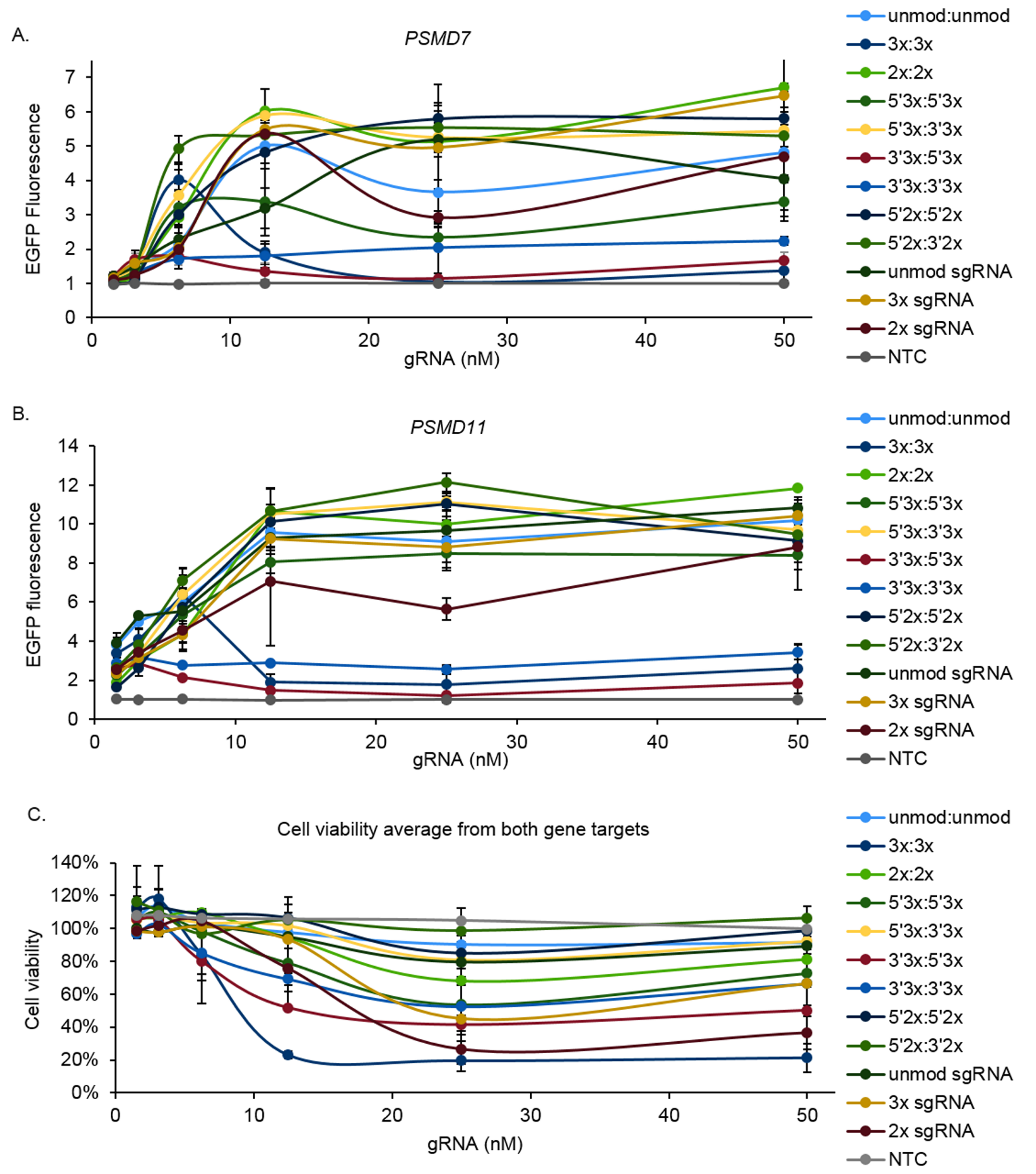

Supplement: S3 Fig — Gene editing efficiency of unmodified (unmod) and modified crRNA:tracrRNA or sgRNA showed similar levels of gene editing efficiencies (< 1.5-fold difference) measured by EGFP fluorescence from knockout of a proteasome component, PSMD7 (A.) or PSMD11 (B.), at multiple concentrations when transfected at 1.5625 nM to 50 nM at 2-fold increments into a stably expressing Cas9 U2OS cell line. Error bars are representative of biological triplicates. C. Average cell viability of unmodified or modified guide RNAs for two genes (PSMD7 and PSMD11) resulted in a significant decrease in cell viability (< 60%) for some modification patterns at concentrations higher than 6.25 nM. NTC = Non-targeting control. Error bars are representative of the average of biological triplicates of two genes with the same modification pattern in one experiment. (TIF) [file pone.0188593.s005.tif]
